# Halofuginone Has Anti-Proliferative Effects in Acute Promyelocytic Leukemia by Modulating the Transforming Growth Factor Beta Signaling Pathway

**DOI:** 10.1371/journal.pone.0026713

**Published:** 2011-10-28

**Authors:** Lorena L. de Figueiredo-Pontes, Patricia A. Assis, Bárbara A. A. Santana-Lemos, Rafael H. Jácomo, Ana Sílvia G. Lima, Aglair B. Garcia, Carolina H. Thomé, Amélia G. Araújo, Rodrigo A. Panepucci, Marco A. Zago, Arnon Nagler, Roberto P. Falcão, Eduardo M. Rego

**Affiliations:** 1 Hematology Division of the Department of Internal Medicine, Medical School of Ribeirão Preto, University of São Paulo, Ribeirão Preto, São Paulo, Brazil; 2 Hematology Division of the Department of Internal Medicine, Medical School of the University of Brasília, Brasília, Brazil; 3 Hematology Division and Cord Blood Bank, Chaim Sheba Medical Center, Tel Aviv University, Tel Hashomer, Israel; French National Centre for Scientific Research, France

## Abstract

Promyelocytic leukemia-retinoic acid receptor alpha (PML-RARα) expression in acute promyelocytic leukemia (APL) impairs transforming growth factor beta (TGFβ) signaling, leading to cell growth advantage. Halofuginone (HF), a low-molecular-weight alkaloid that modulates TGFβ signaling, was used to treat APL cell lines and non-obese diabetic/severe combined immunodeficiency (NOD/SCID) mice subjected to transplantation with leukemic cells from *human chorionic gonadotrophin-PML-RARα* transgenic mice (TG). Cell cycle analysis using incorporated bromodeoxyuridine and 7-amino-actinomycin D showed that, in NB4 and NB4-R2 APL cell lines, HF inhibited cellular proliferation (*P*<0.001) and induced apoptosis (*P* = 0.002) after a 24-hour incubation. Addition of TGFβ revealed that NB4 cells were resistant to its growth-suppressive effects and that HF induced these effects in the presence or absence of the cytokine. Cell growth inhibition was associated with up-regulation of TGFβ target genes involved in cell cycle regulation (*TGFB*, *TGFBRI*, *SMAD3*, *p15*, and *p21*) and down-regulation of *MYC*. Additionally, TGFβ protein levels were decreased in leukemic TG animals and HF *in vivo* could restore TGFβ values to normal. To test the *in vivo* anti-leukemic activity of HF, we transplanted NOD/SCID mice with TG leukemic cells and treated them with HF for 21 days. HF induced partial hematological remission in the peripheral blood, bone marrow, and spleen. Together, these results suggest that HF has anti-proliferative and anti-leukemic effects by reversing the TGFβ blockade in APL. Since loss of the TGFβ response in leukemic cells may be an important second oncogenic hit, modulation of TGFβ signaling may be of therapeutic interest.

## Introduction

Transforming growth factor beta (TGFβ) is a cytokine that regulates multiple cellular responses, including inhibition of cell proliferation and induction of differentiation, senescence, and apoptosis [1], [2]. Its actions are mediated by binding to the serine/threonine kinase receptor TβRII that recruits and activates TβRI, which in turn phosphorylates downstream targets. These include the proteins SMAD2 and SMAD3, which translocate to the nucleus in a complex with the common mediator SMAD4 to regulate transcription of target genes [3], [4]. The tumor suppressor responses of TGFβ are essential for maintaining homeostatic control of normal cell growth and cells in the early phases of tumorigenesis. Among the TGFβ-mediated effects in premalignant cells are the suppression of c-Myc expression [5] and the induction of the cell cycle inhibitors p15 and p21. Although these actions imply a tumor suppressor role for TGFβ, its effects are both cell- and context-dependent. In that regard, Siegel *et al.* have shown that activation of TGFβ delays the appearance of primary mammary tumors, and mice deficient in TGFβ signaling are prone to earlier tumor development, suggesting that the tumor suppressor response of TGFβ is important in the early stages of tumorigenesis. In contrast, mice expressing an activated TGFβ receptor exhibited increased metastatic lung foci, consistent with a pro-oncogenic effect of this pathway in late-stage disease [6]. In addition, advanced disease is accompanied by increased expression and activation of the ligand but decreased TGFβ responsiveness, thus facilitating tumor cell growth [7].

Deregulation of TGFβ signaling may alter hematopoiesis, causing a predisposition to leukemia. In contrast to solid tumors, mutations in SMAD genes are rare in leukemia and disruption of TGFβ responsiveness is commonly secondary to either (a) altered transcription, as described in acute myeloid leukemia with translocation t(8;21), in which the AML1/ETO chimeric protein represses transcription of TGFβ-responsive genes [8] or (b) disruption of TGFβ target gene expression such as the cell cycle regulators c-Myc, p15 and p21, which are commonly associated with leukemogenesis [9].

The role of TGFβ in leukemogenesis has been recently studied in acute promyelocytic leukemia (APL), a distinct subtype of acute myeloid leukemia (AML) associated with t(15;17) and expression of the promyelocytic leukemia-retinoic acid receptor alpha (PML-RARα) hybrid protein. A gene expression study using microarrays has revealed that TGFβ was downregulated in APL compared with most non-APL samples [10]. In contrast, Raza *et al.* have described elevated TGFβ protein expression by immunohistochemistry in bone marrow biopsies of 23 APL patients [11]. Lin *et al.* have demonstrated that the cytoplasmic isoform of PML (cPML) is essential for TGFβ signaling and *Pml*-null primary cells are resistant to TGFβ-dependent growth arrest, induction of cellular senescence, apoptosis, phosphorylation of Smad2/3, and induction of *p15* and *p21* expression. Restoration of cPML fully rescued these defects [12]. Since cPML function is impaired in APL blasts, through the formation of cPML/PML-RARα heterodimers, the authors hypothesized that this would be the molecular mechanism of resistance to TGFβ anti-proliferative responses [13].

To better characterize the deregulation of the TGFβ pathway in APL and to determine its potential as a therapeutic target, we took advantage of the human chorionic gonadotrophin (hCG)-PML/RARα transgenic model and analyzed the effects of halofuginone (HF; *dl*-trans-7-bromo-6-chloro-3-[3-(3-hydroxy-2piperidyl)acetonyl]-4(3*H*)-quinazolinone hydrobromide), which is a low-molecular-weight alkaloid that has been shown to modulate TGFβ signaling. In several cultured cell lines, this drug decreased TGFβ-induced phosphorylation of SMADs 2 and 3 and induced expression of inhibitory SMAD7 mRNA [14]. HF also reduced tumor growth in *in vivo* models of pheochromocytoma [15], brain tumors [16], and hepatocellular carcinoma [17]. The effects of HF in hematopoietic malignancies have not been previously described. Our results demonstrate that HF treatment induces anti-proliferative and pro-apoptotic effects, up-regulates TGFβ target gene expression, and significantly reduces the leukemic burden *in vivo*.

## Materials and Methods

### Ethics Statement

This study was approved by the *Research Ethics Committee of the University Hospital of the Medical School of Ribeirão Preto, University of São Paulo*, process number 3865/2005). Experiments using mice were conducted according to national guidelines for the care and use of laboratory animals (*Brazilian College of Animal Experimentation*) and was approved by the institutional *Animal Experimentation Ethics Committee* (protocol number 088/2007).

### Cell culture

NB4, a permanent cell line harboring t(15;17) [18], and its derivative NB4-R2, in which all-trans retinoic acid (ATRA)-unresponsiveness is associated with a point mutation in the retinoid-binding domain of PML-RARα [19], were used for *in vitro* assays. Cells were cultured in RPMI 1640 with 10% heat-inactivated fetal calf serum (FCS; Gibco BRL, UK) and maintained at 37°C in a CO_2_-humidified incubator.

### Treatment of APL cell lines with HF

HF was kindly provided by Prof. Arnon Nagler (Chaim Sheba Medical Center, Tel Hashomer, Israel). Stock solutions of 1 mg/mL were kept at −80°C until use. Subsequently, working solutions of 10 ng/µL were freshly prepared by diluting the stock solution with autoclaved water (for cell culture assays) or 0.9% NaCl (for *in vivo* studies).

Cell suspensions containing 5×10^5^ cells/mL of culture were treated with increasing doses of HF (6.25–200 ng/mL), which was directly added to the medium, and then cells were harvested after 24, 48, or 72 hours of incubation as indicated. Cell viability measurements were recorded with an initial minimum viability of at least 95% as determined by the Trypan blue assay. For cell cycle analysis and gene expression studies, NB4 cells were also subjected to concurrent treatment with TGFβ (0.5 ng/mL; Sigma-Aldrich, St. Louis, MO, EUA) as indicated.

### Cell proliferation and apoptosis assays

For the analysis of proliferation and the cell cycle, NB4 and NB4-R2 cells were treated with HF as described above for 24 hours and then were subjected to immunofluorescent staining of incorporated bromodeoxyuridine (BrdU) and 7-amino-actinomycin (7-AAD), followed by flow cytometric analysis using the BrdU Flow Kit (BD Biosciences, San Jose, CA, USA). In this method, BrdU, an analog of the DNA precursor thymidine, is incorporated into newly synthesized DNA by cells entering and progressing through the S (DNA synthesis) phase of the cell cycle. The incorporated BrdU is stained with a specific BrdU allophycocyanin (APC)-conjugated antibody and 7-amino-actinomycin (7-AAD), a dye that binds to total DNA. With this combination, a two-color flow cytometric analysis permits the enumeration and characterization of cells that are actively synthesizing DNA (BrdU incorporation) according to their cell cycle position (*i.e.*, G0/1, S, or G2/M phases defined by 7-AAD staining intensities). Cells were incubated with 10 µM of BrdU during the last 30 minutes of culture, and then processed according to the manufacturer's recommendations. For the NB4 cell line only, TGFβ was added or not to the culture medium to evaluate the tumor suppressive effects of TGFβ.

For the analysis of apoptosis after 24 and 48 hours of HF treatment, NB4 cells that were treated with different concentrations of HF were evaluated using the Trypan blue exclusion assay. Concomitantly, apoptosis was determined using the Annexin V and propidium iodide (PI) binding assay (BD Biosciences), and then analyzed by flow cytometry. Each sample was washed in 1× phosphate-buffered saline (PBS), and then incubated with 5 µL of Annexin V, 5 µL of PI, or both at 4°C for 15 min. Subsequently, 400 µL of binding buffer was added to the samples.

All experiments were performed in triplicate and in each sample, 10,000 events were acquired in a FACSCalibur flow cytometer (BD Biosciences) and analysis was performed using Cell Quest software.

The effective dose at 50% (ED_50_) of HF was calculated based on the inhibition of proliferation in the NB4 cell line as determined by the BrdU incorporation assay. This analysis was performed using CalcuSyn software (Biosoft, Great Shelford, UK).

### Analysis of TGFβ target gene expression by real-time polymerase chain reaction (PCR)

NB4 and NB4-R2 cells were treated as above for 24 or 72 hours, and wherever indicated, TGFβ (0.5 ng/mL) was added 1 hour before HF to the culture. Total RNA was isolated using Trizol reagent (Invitrogen, Carlsbad, CA, USA) according to the manufacturer's instructions. Reverse transcription of 500 ng of RNA was performed using the cDNA High Capacity Archive kit (Applied Biosystems, Foster City, CA, USA). Subsequently, the mRNA expression of TGFβ target genes that are involved in cell cycle regulation (*TGFB*, *TGFBRI*, *p15*, *p21*, *SMAD3*, and *MYC*) was evaluated by real-time PCR using the Taqman method. All the cDNA samples were diluted five times and were processed in duplicate. The PCR amplification was performed in 40 cycles, using the Taqman PCR master mix, in an SDS (Sequence Detection System) 5700 plataform connected to a 7300 Real-Time PCR System (Applied Biosystems). The probes used for amplification were synthesized using the Assay-on-Demand System (Applied Biosystems) with the following GeneBank sequences: *TGFβ* (NM_003236, FAM-TGAACCAAGGAGACGGAATACAGGG-NFQ), *TGFβRI* (NM_004612.2, FAM-TGGCAGAGCTGTGAAGCCTTGAGAG-NFQ), *SMAD3* (NM_005902.3, FAM-GGGAGCGGAGTACAGGAGACAGACT-NFQ), *p15* (NM_006428.3, FAM-CCAACAACGACAAGCTCTCCAAGAG-NFQ), *p21* (NM_000389.2, FAM-GGCAGACCAGCATGACAGATTTCTA-NFQ), and *MYC* (NM_002467.3, FAM-AACCAGCAGCCTCCCGCGACGATGC-NFQ). The expression of the *glyceraldehyde-3-phosphate dehydrogenase* (*GAPDH*) housekeeping gene was determined using the PDAR reagent (Pre-Developed Assay Reagent; Applied Biosystems) and was used to normalize the data. The 2^−ΔΔCT^ method was used in the analysis of the PCR data and the relative gene expression in a particular sample was determined as follows: relative amount of target = 2^−ΔΔCT^ value.

### Detection of TGFβ protein expression

After treatment with HF as described above, total protein extracts were obtained according to Schreiber *et al.* [20]. Briefly, NB4 cells were washed twice in cold PBS, lysed with lysis buffer (20 mM Tris–HCl, pH 7.5, 150 mM NaCl, 1 mM Na_2_EDTA, 1% Triton, 2.5 mM sodium pyrophosphate, 1 mM β- glycerophosphate, 1 mM Na_3_VO_4_, 1 mg/mL leupeptin) containing a protease inhibitor mixture P8340 (Sigma, St. Louis, MO, USA) and homogenized in a Dounce system (model D-130, Biosystems, Brazil) for one minute on ice. Lysates were centrifuged at 20,000× g for 30 minutes at 4°C and the supernatants were removed. Protein concentration was determined by the Bradford's method [21]. Proteins were submitted to SDS–PAGE and a total of 30 µg of protein from each sample was transferred to polyvinylidene fluoride (PVDF) membranes (GE Lifesciences, Pittsburgh, PA, USA) [22]. Membranes were blocked with 5% non-fat dry milk in 0.1% Tween-TBS and incubated with the specific antibodies. Mouse anti-β-actin was purchased from Santa Cruz Biotechnology (California, USA). Rabbit anti-TGFβ, anti-TGFβ receptor II, anti-Smad3 and horseradish peroxidase-conjugated goat anti-rabbit IgG secondary antibody were purchased from Cell Signaling (Beverly, MA, USA), and goat anti-mouse IgG secondary antibody from GE Lifesciences (Pittsburgh, PA, USA). The antibody-protein complex was detected using the ECL Western Blotting Detection Reagents (GE Lifesciences).

### HF treatment in an APL transplant model

To analyze *in vivo* effects of HF, irradiated immunodeficient non-obese diabetic/severe combined immunodeficiency (NOD/SCID) mice were injected with leukemic cells from hCG-PML-RARα transgenic mice (TM). The TM were kindly provided by Prof. Pier Paolo Pandolfi (Beth Israel Deaconess Medical Center, Harvard Stem Cell Institute, Boston, MA, USA) and their generation has been described elsewhere [23]. Notably, in this transgenic model, a lethal form of leukemia that closely resembles human APL occurs after a long pre-leukemic phase (12–15 months) and affects only 10–15% of the TM [24]. We have established a transplant model, in which all animals develop leukemia after 14 days from the transplant. Briefly, leukemic cells, previously maintained at −80°C, were thawed and suspended in RPMI 1640 with 10% FCS. After Trypan blue exclusion testing and 12 hours after sublethal cobalt irradiation with 250 cGy, 2×10^6^ viable cells were intravenously injected into the ocular plexus of CB17-Prkdc^scid^/J 10- to 12-week-old NOD/SCID mice (The Jackson Laboratory, Bar Harbor, Maine, USA). Animals were maintained under pathogen-free conditions and received autoclaved food and water *ad libitum*. Experiments were conducted according to institutional and national guidelines for the care and use of laboratory animals

The definition of the dose and mode of administration of HF was based on previous reports of the *in vivo* use of the drug in solid tumors and fibrosis models [14], [15], [17], [25], [26], [27], [28], and pilot experiments were performed to confirm leukemic infiltration in transplanted animals and to test the efficacy and toxicity of HF. Twenty-four hours after the transplant procedure, NOD/SCID mice received treatment with vehicle only (0.9% NaCl; *n* = 5) or 150 µg/kg/day HF (*n* = 5) as an intraperitoneal injection for 21 consecutive days. At the end of the experiment (day 21), mice were sacrificed under ketamine anesthesia after being subjected to a cardiac puncture to obtain peripheral blood (PB) samples. Non-leukemic, age-matched NOD/SCID were used as controls (*n* = 3). Animals were maintained under pathogen-free conditions and received autoclaved food and water *ad libitum*.

### Analysis of the hematological and molecular responses to HF *in vivo*

For monitoring PB counts, mice were bled from the tail before transplantation and 10 days after the beginning of HF injections. Automated counts (hemoglobin, white blood cells, and platelets) were performed using a T-890 Coulter cell counter (Coulter Corporation, Hialeah, FL, USA), and differential counts were obtained from Leishman-Wright-Giemsa-stained smears. In addition, after euthanasia, bone marrow (BM) cells were obtained by flushing the bone cavities of femurs and tibiae with RPMI 1640 containing 10% FCS. Cells were washed once, and then the pellet was resuspended in PBS at a concentration of 10^6^/mL. Approximately 10^5^ cells were used for cytospin slide preparation and staining with Leishman-Wright-Giemsa, and then 10^6^ cells were used for DNA extraction and PCR analysis to detect PML-RAR**α**, as previously described by van Dongen [29].

For morphological analysis of PB and bone marrow slides, a minimum of 100 PB and 200 BM cells were counted, and then myeloid cells were classified as immature, intermediate, or mature, according to the Bethesda proposals for classification of non-lymphoid hematopoietic neoplasms in mice [30]. Because extensive spleen infiltration was observed in leukemic animals, the relation between the spleen and body weight was also assessed to further quantify the hematological response to HF.

### Quantification of TGFβ in the serum of leukemic mice treated with HF

Serum and BM samples of transplanted and control NOD/SCID mice were obtained after euthanasia, as previously described, and used to quantify TGFβ using the enzyme-linked immunosorbent assay (ELISA). Serum samples were stocked at −80°C until use. BM cells were washed twice with 1× PBS and total protein extracts were prepared by suspending cell pellets in a lysis buffer (20 mM Tris pH 7.5, 150 mM NaCl, 1 mM EDTA, 1 mM EGTA, 1% Triton X-100, 2.5 mM sodium pyrophosphate, and 1 mM PMSF) supplemented with a cocktail of protease and phosphatase inhibitors (Sigma Aldrich), and then subjecting the samples to three cycles of sonication. The resulting lysates were centrifuged at 13000 rpm for 10 minutes at 4°C, and then supernatants (a minimum total protein of 1 µg) were stocked at −20°C until use. Subsequently, serum and BM protein samples were subjected to activation of latent TGFβ1 to immunoreactive TGFβ by acidification, and then used in a sandwich ELISA assay using the Quantikine TGFβ1 Immunoassay kit (R&D Systems, Minneapolis, MN, USA), according to the manufacturer's recommendations. The optical density was determined using a microplate reader set at an absorbance of 450 nm and a standard curve was generated to report the results of TGFβ quantification in pg/mL.

### Statistical analysis

The effects of HF on APL cell lines regarding cell proliferation, apoptosis, BCL-2 expression, and transcription of TGFβ target genes were analyzed by multivariate analysis using the mixed linear model. Comparisons simultaneously included the following variables: treatment with variable doses of HF, cell type (either NB4 or NB4-R2), and addition of TGFβ. For *in vivo* experiments, differences in blood cell counts, percentage of immature cells, comparisons of the spleen/body weight ratio, and TGFβ quantification in leukemic and control mice were evaluated by analysis of the variance (ANOVA) between groups followed by the Bonferroni's correction post-test. In all comparisons, a significance level of *P*<0.05 was considered to be significant. Statistical analyses were performed using SPSS 13.0 software.

## Results

### HF exerts anti-proliferative actions on APL

**Figure 1:**
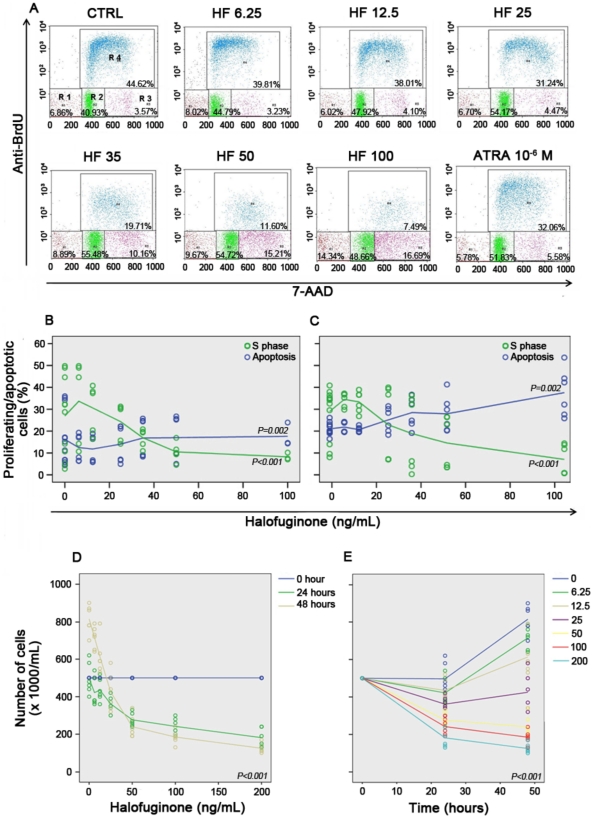
Halofuginone (HF) exerts antiproliferative effects in acute promyelocytic leukemia (APL) cell lines NB4 and NB4-R2. (*A*) Representative example of one out of three experiments of flow cytometric analysis of bromodeoxyuridine (BrdU; y-axis) and 7-amino-actinomycin (7-AAD; x-axis) staining of NB4 cells incubated with increasing doses of HF or all-*trans* retinoic acid (ATRA, as a control), showing HF-induced growth arrest at the G1/S transition. Cell subpopulations were identified as R1 (sub G0/G1, apoptotic cells), R2 (G0/G1), R3 (G2+M), and R4 (S phase). (*B*–*C*) Cell cycle status of NB4 (*B*) and NB4-R2 (*C*) cells after treatment with increasing doses of HF and BrdU incorporation. Percentage of cells in S phase or apoptosis is identified by green or blue lines, respectively. Results confirmed that, in both cell lines, HF inhibited cell proliferation (*P*<0.001) and caused apoptosis (*P* = 0.002), although the pro-apoptotic effect in NB4 cells was visually less evident than the one observed in NB4-R2. (*D*–*E*) Analysis of the number of viable NB4 cells by Trypan blue exclusion according to the incubation time (*P*<0.001) with various doses of HF (*P*<0.001), indicated by the right-sided legend. Forty-eight hours after incubation with doses greater than 50 ng/mL of halofuginone, the number of viable NB4 cells significantly reduced.

**Figure 2:**
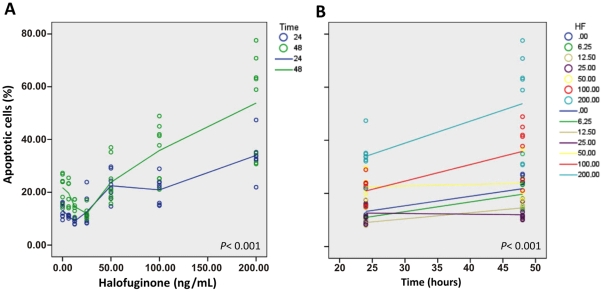
Halofuginone (HF) induces apoptosis in a dose-dependent manner in acute promyelocytic leukemia (APL) cell line NB4. (*A*, *B*) Analysis of the number of apoptotic NB4 cells by Annexin V and propidium iodide binding assay according to the incubation time (*P*<0.001) with various doses of halofuginone (*P*<0.001), indicated by the right-sided legend. Apoptosis was more pronounced after the 48-hour treatment (*A*) and occurred particularly when doses greater than 100 ng/mL were used (*B*).

Cell cycle analysis, evaluated by the immunofluorescent staining of incorporated BrdU and 7-AAD, showed that, in both NB4 and NB4-R2 cell lines, HF inhibited cellular proliferation (*P*<0.001) and induced apoptosis (*P* = 0.002) in a dose-dependent manner. Increasing doses of HF blocked cell cycle progression at the G1/S transition (Fig. 1*A–C* and Figure S1). In addition, doses greater than 35 ng/mL of halofuginone also induced an accumulation of cells in G2+M. Notably, NB4-R2 cells were more susceptible to HF than NB4 cells (*P*<0.001) (Fig. 1*A–C* and Figure S1). Similar results were detected using the Trypan blue exclusion test. Compared to control samples, treatment with 50 ng/mL HF reduced NB4 cell viability by 44.3% (40–46.77%) and 70.5% (62.3–72.8%) after 24 and 48 hours of treatment, respectively. Indeed, no increase in viable cells numbers was observed after 24 hours of treatment with more than 50 ng/mL HF (Fig. 1*D*–*E*). Simultaneously, doses of HF greater than 50 ng/mL induced initial and late-stage apoptosis after 24 hours of treatment as determined by Annexin V and PI binding assays and flow cytometric analysis (*P*<0.001). In addition, after 48 hours of incubation, HF induced a 1.6- and 2.5-fold increase in apoptosis at 100 and 200 ng/mL, respectively (Fig. 2*A–B*).

**Figure 3:**
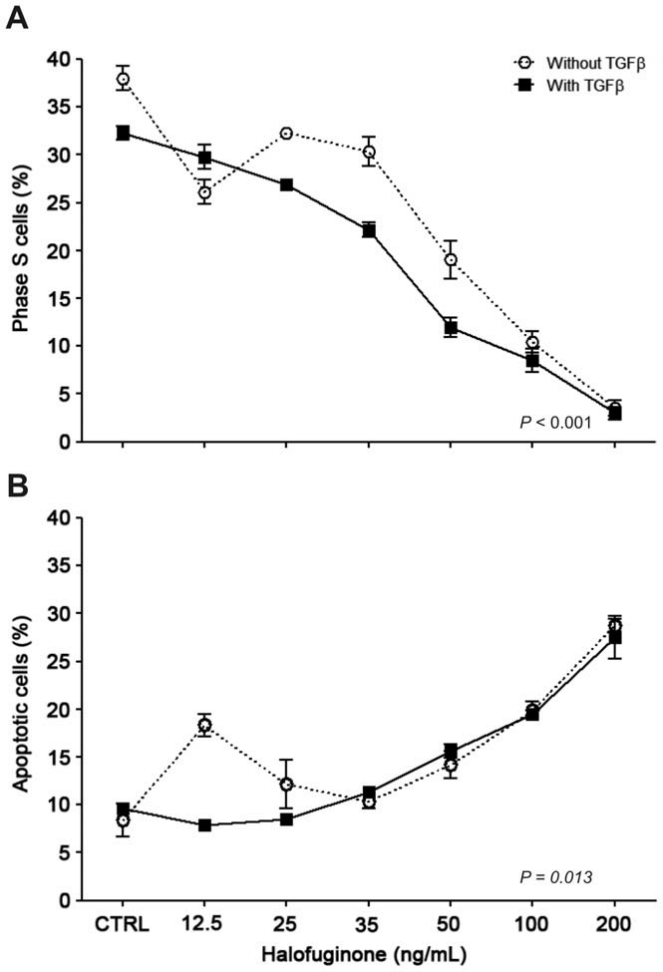
Treatment of APL cells with halofuginone (HF) results in growth arrest (*A*) and apoptosis (*B*) regardless of TGFβ exogenous stimulus. Cell cycle effects were determined by the BrdU incorporation assay. Continuous and dotted lines represent the effects of HF in the absence or presence of exogenous TGFβ in cell cultures, respectively. The multivariate analysis by the mixed linear model revealed that addition of TGFβ is statistically relevant (*P*<0.001) for cell proliferation and apoptosis (*P* = 0.013). Regarding the latter, the percentage of apoptotic cells was similar in samples treated with 25–200 ng/mL of HF irrespective of TGFβ addition. Therefore, the significant difference reflects the results detected with 12.5 ng/mL of halofuginone. The effective dose at 50% (ED_50_) of HF for the inhibition of proliferation was 18 ng/mL, whereas, in the presence of TGFβ, the ED_50_ was reduced to 10 ng/mL, thus suggesting a potential additive anti-proliferative action of TGFβ and HF. Data represent the results of three independent experiments.

When TGFβ was added to NB4 cell cultures with HF at concentrations of 25–50 ng/mL, a significant increase (*P*<0.001) in growth inhibition was observed, suggesting an additive effect between the two molecules. At doses ≥100 ng/mL of HF, less than 10% of the cells were in S phase, suggesting that a maximum effect was reached (Fig. 3*A*). Regarding apoptosis, the effect of HF was similar in the presence or absence of TGFβ (Fig. 3*B*), except for the treatment with 12.5 ng/mL of HF. Although the multivariate analysis by the mixed linear model revealed that addition of TGFβ is statistically relevant for apoptosis (*P* = 0.013), this was due to the results detected with 12.5 ng/mL of HF.

The ED_50_ of HF for the inhibition of proliferation after 24 hours of incubation was calculated based on the percentage of cells in S phase. Without concomitant treatment with TGFβ, the ED_50_ of HF was 18 ng/mL (95% confidence interval (95% CI): 15.5–20.8 ng/mL; R^2^: 0.99). In the presence of TGFβ, the ED_50_ was 10 ng/mL (95% CI: 7.45–15.7 ng/mL; R^2^: 0.98), thus corroborating the potential additive anti-proliferative action of TGFβ and HF.

### Halofuginone up-regulates the expression of TGFβ target genes and TGFβ protein

**Figure 4:**
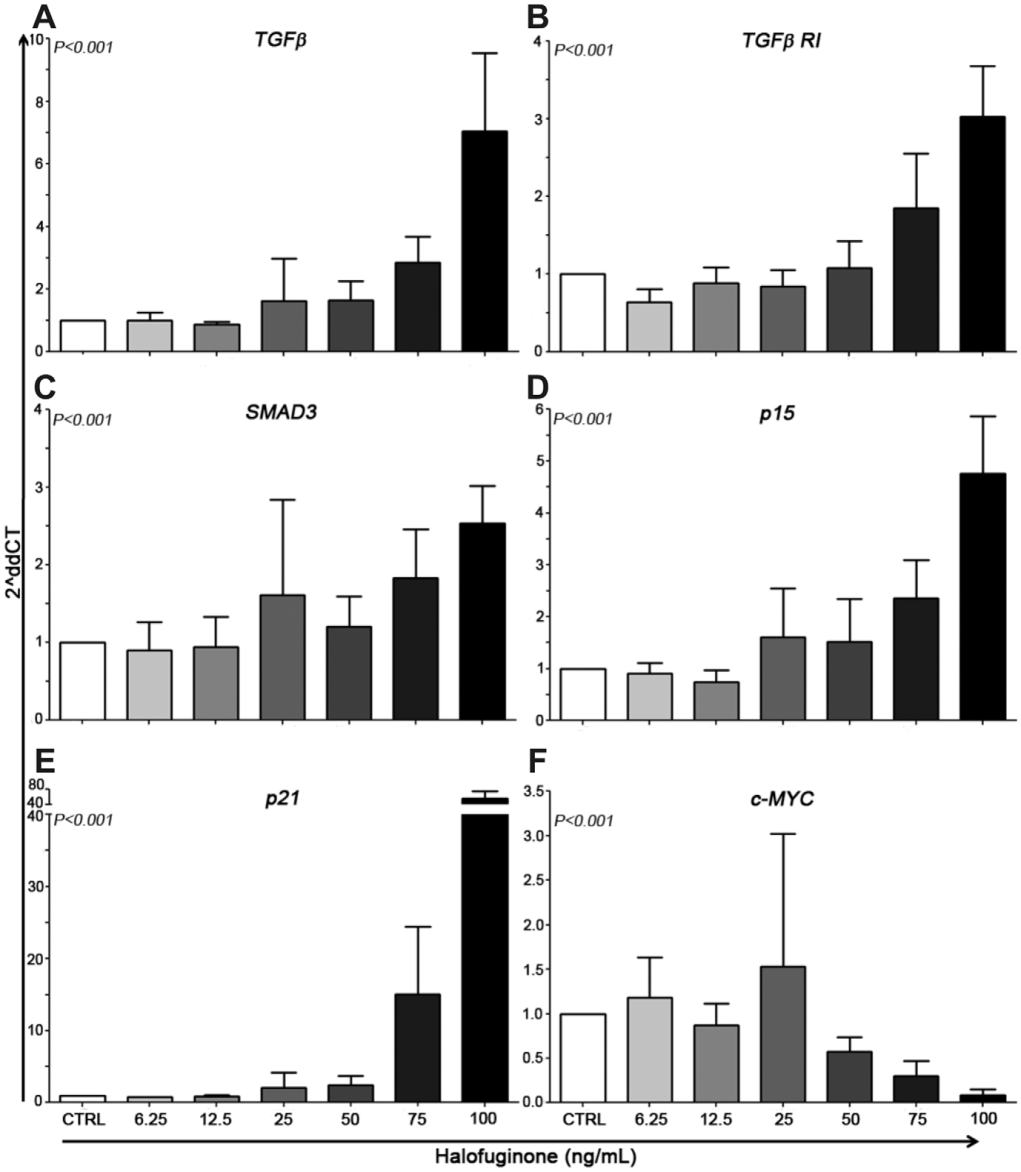
Halofuginone (HF) induces activation of TGFβ signaling in APL cell lines. (*A*–*F*) Real-time PCR analysis of TGFβ and its target genes in NB4 cells treated with increasing doses of HF, showing up-regulation of *TGFβ* (*A*), *TGFβRI* (*B*), *SMAD3* (*C*), *p15* (*D*), *p21* (*E*), and repression of *MYC* (*F*).

**Figure 5:**
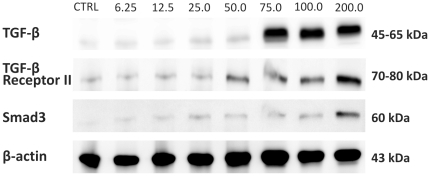
Halofuginone (HF) up-regulates the expression of TGFβ, TGFβ receptor II and Smad3 protein in the NB4 APL cell line. Western blot analysis shows that increasing doses of HF (6.25–200 ng/mL) induced the expression of TGFβ and its mediators after 72 hours of incubation. CTRL: control.

HF dose-dependent up-regulation of *TGFβ* (*P*<0.001), *TGFβRI* (*P*<0.001), *SMAD3* (*P*<0.001), *p15* (*P*<0.001), and *p21* (*P*<0.001) gene expression was detected after 72 hours of incubation. In addition, treatment resulted in the down-regulation of *MYC* (*P*<0.001) with concentrations higher than 25 ng/mL (Fig. 4*A*–*F*). The expression of TGFβ protein was evaluated by Western blotting and HF induced a dose-dependent increase in TGFβ, TGFβ receptor II and Smad3 expression in NB4 cells (Fig. 5).

**Figure 6:**
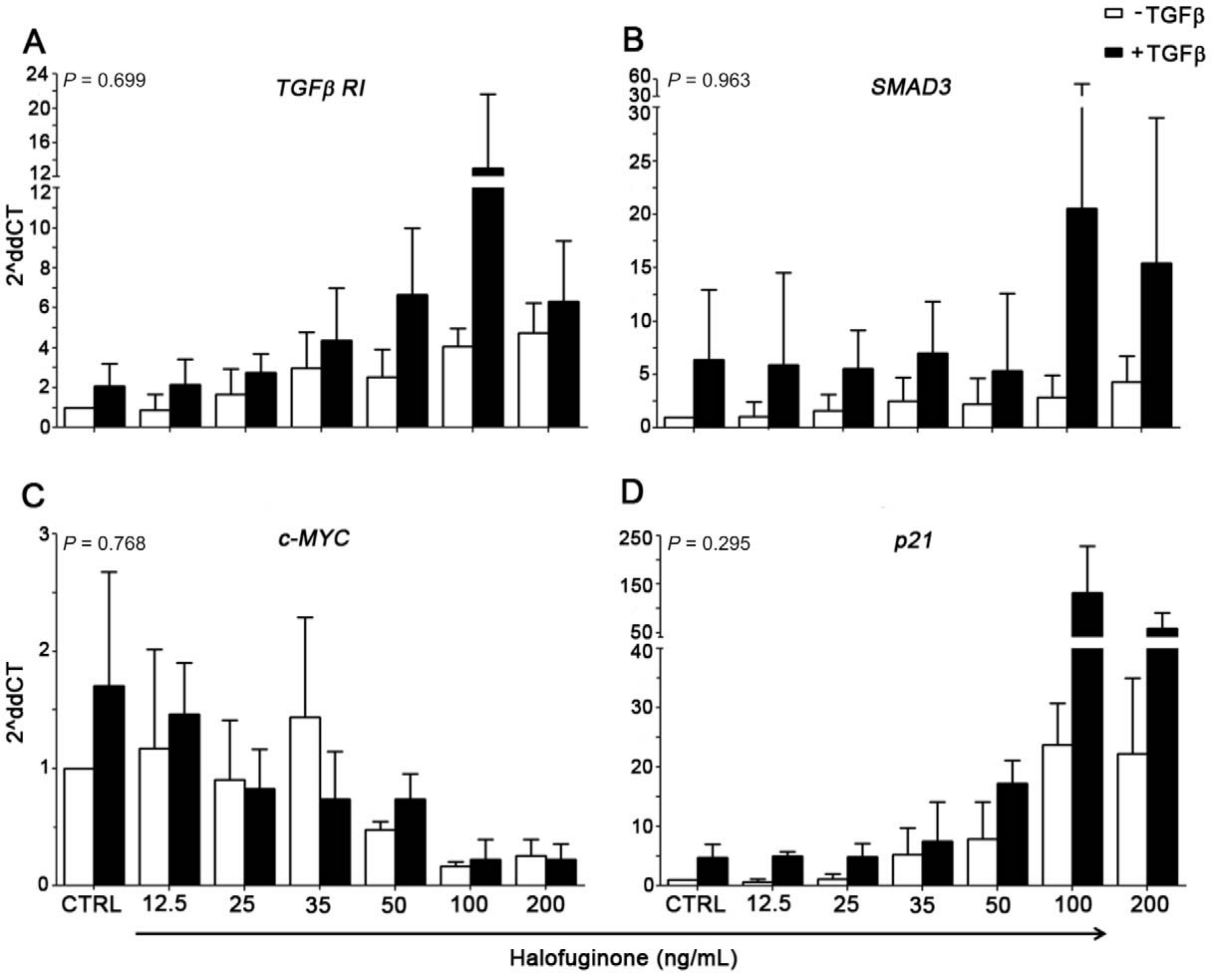
Halofuginone (HF) induces activation of TGFβ signaling in APL cells regardless of exogenous TGFβ addition. (*A*–*D*) Real-time PCR analysis in the presence or absence of exogenous TGFβ, showing similar effects of HF in NB4 cells: (A) *TGFβRI* (*P* = 0.699); (B) *SMAD3* (*P* = 0.963); (C) *MYC* (*P* = 0.768), and (D) *p21* (*P* = 0.295).

To verify that these effects were caused by HF treatment and not a late cellular response to cell growth inhibition, some genes were selected for a 24-hour HF incubation and real time PCR analysis, including the addition of exogenous TGFβ to NB4 cultures. Consistent with the 72-hour treatment assay, after 24-hour treatment, HF significantly induced the transcription of TGFβ target genes (*TGFβRI* and *p21*) and repressed *MYC* expression in NB4, regardless of the addition of exogenous TGFβ (Fig. 6). Indeed, as analyzed using the mixed linear model, the results were similar in the presence or absence of TGFβ for *TGFβRI* (*P* = 0.699), *SMAD3* (*P* = 0.963), *p21* (*P* = 0.295), and *MYC* (*P* = 0.768).

### HF demonstrates anti-leukemic effects in the *in vivo* model of APL

Table 1 shows that HF treatment for 21 days resulted in an increase to normal hemoglobin levels and platelet counts in leukemic mice. The WBC was significantly lower in leukemic mice treated with HF compared with untreated controls. However, the cytomorphological analysis of *Leishman*-stained PB smears demonstrated that HF treatment reduced the percentage of immature cells in the bone marrow (66.3±17.9% versus 27±9.3%; *P*<0.01) and peripheral blood samples (36.5±21.04% versus 15±5.09%; *P* = 0.06), although blasts were still detectable (Table 1). PML-RAR**α** detection by PCR confirmed the engraftment of leukemic cells in all transplanted animals. As expected by the morphological analysis of the bone marrow, samples obtained after treatment tested positive for PML-RARα.

**Table 1:** Peripheral blood cell counts, percentage of immature cells, and spleen/body weight ratio of wild-type control mice (WT; n = 3) and NOD/SCID leukemic mice that were treated with halofuginone (HF) (LEUK-HF; n = 5) or left untreated (LEUK; n = 5).

	WT	LEUK	LEUK-HF
Hb (g/dL)	15.3±0.6	9.6±1.67	12.0±1.40^*^
WBC (×10^3^/µL)	2.33±0.57	20.6±21.9	4.2±3.89^**^
Platelets (×10^3^/µL)	1160.0±215.9	552±83.2	932.0±122.5^***^
PB blasts (%)	3.6±1.52	36.5±21.04	15.0±5.09^†^
BM blasts (%)	-	66.3±17.9	27±9.3^†^
Spleen/body weight ratio	0.003±0.0002	0.012±0.004	0.006±0.001

Immunodeficient NOD/SCID mice were injected with leukemic cells from hCG-PML-RARα transgenic mice after sublethal irradiation and, 24 hours later, were treated with vehicle (LEUK; *n* = 5) or 150 µg/kg HF (LEUK-HF; *n* = 5) as an intraperitoneal injection for 21 consecutive days. HF treatment resulted in the reduction of white blood cell counts (WBC), increase in hemoglobin (Hb) levels and platelets count to normal values, reduction of the blast percentage in peripheral blood (PB) and bone marrow (BM), and reduction of the spleen size in leukemic transplanted animals. Results are shown as average values ± standard deviation. The differences of cell counts in leukemic animals treated with HF or not was significant, when analyzed by ANOVA and Bonferroni's post test (^*^ *P*<0.05, ^**^ *P*<0.01, ^***^ *P*<0.001, ^†^ *P*<0.01 for Hb, WBC, platelets and PB/BM blasts, respectively).

Since extensive spleen infiltration has been observed in the hCG-PML-RARα mouse model [23], the relation between the spleen and body weight was assessed to further quantify the hematological response to HF. Compared with NOD/SCID wild-type (WT) control mice, as expected, leukemic animals showed a higher spleen/body weight ratio (*P*<0.001). Treatment with HF resulted in a significant reduction of the spleen/body weight ratio (*P*<0.05; Table 1). In addition, morphological analysis of *Leishman*-stained slides of spleen imprints confirmed the infiltration of immature cells resembling promyelocytes in leukemic animals and demonstrated normal cellular distribution in the HF-treated mice.

### HF reverses TGFβ inhibition in PML-RARα leukemic mice

**Figure 7:**
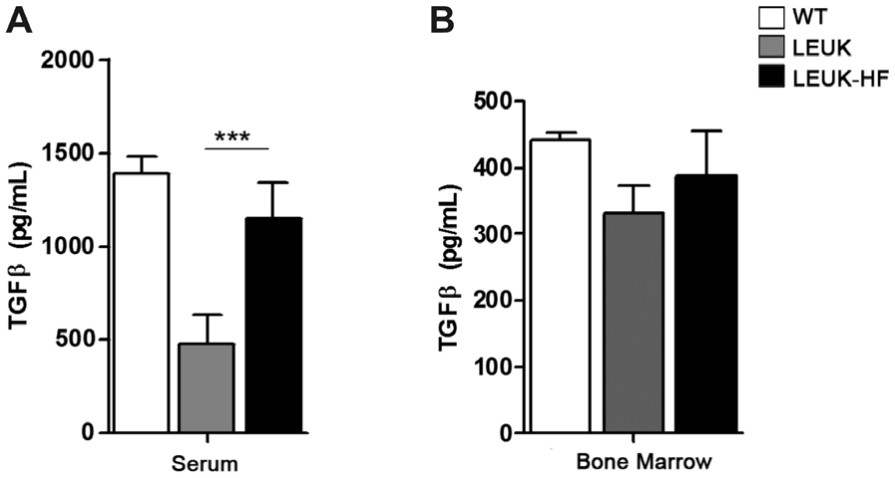
TGFβ quantification in serum (A) and bone marrow (B) of leukemic NOD/SCID mice treated with halofuginone (HF). As determined by enzyme-linked immunosorbent assay, TGFβ serum levels were lower in leukemic transplanted mice (LEUK) compared with wild-type (WT) controls (*P*<0.001), and HF increased TGFβ to levels similar to those in controls.

TGFβ quantification in serum samples showed lower levels of the cytokine in leukemic mice compared with WT controls (*P*<0.0001). In animals that received HF treatment, TGFβ levels were similar to those in controls, suggesting that the drug could reverse TGFβ inhibition in PML-RARα leukemic mice. When TGFβ protein expression was verified in the bone marrow lysate, a similar tendency of TGFβ down-regulation in leukemic animals was observed, although the data were not statistically significant (Fig. 7).

## Discussion

The present study demonstrates for the first time that HF has anti-leukemic properties, reducing tumor growth and inducing apoptosis *in vitro* and *in vivo*. A few studies have reported that HF inhibits angiogenesis *in vitro* [31] and T cell activation [32], with doses ranging from 5 to 400 ng/mL. Previous studies regarding HF pharmacokinetics have suggested that intraperitoneal delivery of a 1.5-mg/kg dose in mice produced plasma concentrations between 173 and 209 ng/mL at 10 minutes after administration, with a 100% bioavailability and rapid distribution of the drug to all tissues, except the brain. In addition, HF doses greater than 1.5 mg/kg proved excessively toxic to mice [33]. *In vivo* studies using HF have reported significant anti-tumoral effects of the drug with intraperitoneal doses varying from 50 to 300 µg/kg with no significant toxicity. Therefore, the doses we have chosen for both *in vitro* and *in vivo* assays were within the range previously reported to be effective and non-toxic. Importantly, HF has been previously shown to reduce solid tumor growth, but no published data exist on its actions in hematologic diseases.

HF exerted a potent anti-proliferative effect with an ED_50_ of 18 ng/mL (95% CI: 15.5–20.8 ng/mL), and was as effective in NB4 as in NB4-R2 cells. HF-induced cell growth inhibition was associated with dose-dependent up-regulation of TGFβ target genes. Treatment resulted in an increase in the number of TGFβ mRNA transcripts and TGFβ protein expression, which is consistent with the previously demonstrated positive feedback of the pathway [34]. Moreover, the expression of other genes that encode proteins of the TGFβ pathway (including TGFβRI and SMAD3) were modulated by HF treatment. SMAD3, when phosphorylated and released from the receptor complex by TGFβRI, forms a heterodimeric complex with SMAD4, which then translocates to the nucleus to regulate transcription of target genes. Consistent with the activation of the TGFβ pathway induced by HF, our results showed up-regulation of the cyclin-dependent kinase inhibitors *p15* and *p21* and down-regulation of *MYC*, which may have contributed to TGFβ growth inhibition. *MYC* appears to be a very specific target of TGFβ because its transcription is mediated by SMAD3 binding to a responsive element in the promoter of the gene [35], [36]. In addition, our findings agree with the evidence that, upon TGFβ stimulation, down-regulation of *MYC* creates a positive feedback loop that further amplifies *p15* and *p21* expression [1], [2], [37].

HF inhibited proliferation and induced apoptosis in the absence of exogenous TGFβ, but addition of the latter potentiated the effects of HF and resulted in a reduction of the ED_50_ concentration by 45% and further up-regulated the expression of *TGFβRI*, *SMAD3* and *p21*. Of note, as shown in figure 6, the addition of TGFβ alone to NB4 cells was able to up-regulate its target genes. This can be attributed to the TGFβ property of activating its own mRNA expression and protein secretion. Therefore, one could hypothesize that the up-regulation of TGFβ target genes by HF results from a combination of increased secretion of TGFβ and a direct effect of the drug on transcription.

The results obtained with HF treatment in a murine transplant model of APL in NOD/SCID mice reinforced the potential anti-leukemic effects of the drug. Mice transplanted with PML-RARα cells and treated with HF presented hematological remission in peripheral blood, bone marrow, and spleen as determined by blood cell counts and cytological analysis. HF did not induce differentiation of leukemic blasts, and the improvement of hematopoiesis resulted from the decrease of the leukemic burden.

In addition, consistent with the evidence of the downregulation of TGFβ signaling in APL, TGFβ protein levels were decreased in leukemic PML-RARα animals and HF increased TGFβ levels to values very similar to those of control non-leukemic mice. The partial hematological remission observed in HF-treated animals may be associated with the direct induction of the cytokine TGFβ itself, known to play a key role in the regulation of human hematopoietic stem cell quiescence, proliferation and differentiation [38]. Although the effect of TGFβ as a potent negative regulator of hematopoieses may contribute to tumor control, its role in leukemogenesis has been more recently associated with the findings of disruption in TGFβ signaling either by mutational inactivation or down-regulation of its components [13]. Therefore, we have attributed the TGFβ-dependent growth inhibitory effects of HF to the indirect transcription regulation of cell cycle target genes.

In contrast to our findings, previous studies of fibrosis models have shown that HF inhibits TGFβ-induced phosphorylation of SMAD2 and 3, abrogating subsequent signaling [14], [26], [39], [40]. This may be explained by the fact that TGFβ functions are both cell type- and context-dependent. In tumorigenesis, it can switch from tumor suppressor in the premalignant stages to prooncogene at later stages of the disease. We believe that malignant cells of APL have decreased or altered TGFβ-responsiveness and that HF was able to restore TGFβ signaling activation.

Of interest, it has been shown that low doses of HF selectively inhibited the differentiation of the proinflammatory T helper 17 murine and human cells (TH17 cells) by activating a cytoprotective signaling pathway, without directly modulating TGFβ-induced SMAD2 phosphorylation or other lymphocytes responses to TGFβ [41]. Indeed, Wu *et al* demonstrated that TH17 cells and interleukin-17 concentrations were significantly increased in peripheral blood samples from AML patients when compared with those from healthy volunteers, suggesting that TH17 cells may play a role in leukemogenesis [42]. Although not dependent on TGFβ regulation, this effect strengthens the potential of HF as an anti-leukemic drug.

Recent evidence has suggested that deregulation of the TGFβ pathway may play a role in APL pathogenesis. PML-RARα may interrupt the interaction of nuclear PML with the SMAD2/3/4 complex, leading to blockade of TGFβ transcriptional activity [13]. In this context, considering the effects on TGFβ target gene expression, HF could reverse this blockade and prevent cell proliferation in an *in vitro* model of APL. Consistent with the hypothesis of TGFβ blockade in APL, our results did not show significant differences in cell growth inhibition and apoptosis when NB4 and NB4 cells treated with TGFβ were compared, suggesting that APL cells are resistant to the anti-proliferative effects of this cytokine.

We can hypothesize that although the disruption of the TGFβ pathway itself is not sufficient to initiate malignant transformation, the loss of the TGFβ response may be a critical second step that contributes to leukemia progression. In this context, the modulation of TGFβ signaling may have therapeutic interest in APL.

## Supporting Information

Figure S1 **Cell cycle status of NB4 and NB4-R2 cells after treatment with increasing doses of halofuginone.** Cell cycle status according to BrdU incorporation by NB4 (upper graphic) and NB4-R2 (lower graphic) cells after treatment with increasing doses of halofuginone. The multivariate analysis using the mixed linear model confirmed that, in both cell lines, the drug inhibited cell proliferation (*P*<0.001) and caused apoptosis (*P* = 0.002), although the pro-apoptotic effect in NB4 cells was visually less evident than the one observed in NB4-R2.
(TIF)

## References

[1] Siegel PM, Massague J (2003). Cytostatic and apoptotic actions of TGF-beta in homeostasis and cancer.. Nat Rev Cancer.

[2] Shi Y, Massague J (2003). Mechanisms of TGF-beta signaling from cell membrane to the nucleus.. Cell.

[3] Nakao A, Imamura T, Souchelnytskyi S, Kawabata M, Ishisaki A (1997). TGF-beta receptor-mediated signalling through Smad2, Smad3 and Smad4.. EMBO J.

[4] Roberts AB (1999). TGF-beta signaling from receptors to the nucleus.. Microbes Infect.

[5] Chen CR, Kang Y, Massague J (2001). Defective repression of c-myc in breast cancer cells: A loss at the core of the transforming growth factor beta growth arrest program.. Proc Natl Acad Sci U S A.

[6] Siegel PM, Shu W, Cardiff RD, Muller WJ, Massague J (2003). Transforming growth factor beta signaling impairs Neu-induced mammary tumorigenesis while promoting pulmonary metastasis.. Proc Natl Acad Sci U S A.

[7] Roberts AB, Wakefield LM (2003). The two faces of transforming growth factor beta in carcinogenesis.. Proc Natl Acad Sci U S A.

[8] Jakubowiak A, Pouponnot C, Berguido F, Frank R, Mao S (2000). Inhibition of the transforming growth factor beta 1 signaling pathway by the AML1/ETO leukemia-associated fusion protein.. J Biol Chem.

[9] Kim SJ, Letterio J (2003). Transforming growth factor-beta signaling in normal and malignant hematopoiesis.. Leukemia.

[10] Gutierrez NC, Lopez-Perez R, Hernandez JM, Isidro I, Gonzalez B (2005). Gene expression profile reveals deregulation of genes with relevant functions in the different subclasses of acute myeloid leukemia.. Leukemia.

[11] Raza A, Yousuf N, Abbas A, Umerani A, Mehdi A (1992). High expression of transforming growth factor-beta long cell cycle times and a unique clustering of S-phase cells in patients with acute promyelocytic leukemia.. Blood.

[12] Lin HK, Bergmann S, Pandolfi PP (2004). Cytoplasmic PML function in TGF-beta signalling.. Nature.

[13] Lin HK, Bergmann S, Pandolfi PP (2005). Deregulated TGF-beta signaling in leukemogenesis.. Oncogene.

[14] Xavier S, Piek E, Fujii M, Javelaud D, Mauviel A (2004). Amelioration of radiation-induced fibrosis: inhibition of transforming growth factor-beta signaling by halofuginone.. J Biol Chem.

[15] Gross DJ, Reibstein I, Weiss L, Slavin S, Dafni H (2003). Treatment with halofuginone results in marked growth inhibition of a von Hippel-Lindau pheochromocytoma in vivo.. Clin Cancer Res.

[16] Abramovitch R, Itzik A, Harel H, Nagler A, Vlodavsky I (2004). Halofuginone inhibits angiogenesis and growth in implanted metastatic rat brain tumor model–an MRI study.. Neoplasia.

[17] Taras D, Blanc JF, Rullier A, Dugot-Senant N, Laurendeau I (2006). Halofuginone suppresses the lung metastasis of chemically induced hepatocellular carcinoma in rats through MMP inhibition.. Neoplasia.

[18] Lanotte M, Martin-Thouvenin V, Najman S, Balerini P, Valensi F (1991). NB4, a maturation inducible cell line with t(15;17) marker isolated from a human acute promyelocytic leukemia (M3).. Blood.

[19] Duprez E, Benoit G, Flexor M, Lillehaug JR, Lanotte M (2000). A mutated PML/RARA found in the retinoid maturation resistant NB4 subclone, NB4-R2, blocks RARA and wild-type PML/RARA transcriptional activities.. Leukemia.

[20] Schreiber G, Schreiber M (1973). The preparation of single cell suspensions from liver and their use for the study of protein synthesis.. Subcell Biochem.

[21] Bradford MM (1976). A rapid and sensitive method for the quantitation of microgram quantities of protein utilizing the principle of protein-dye binding.. Anal Biochem.

[22] Towbin H, Staehelin T, Gordon J (1979). Electrophoretic transfer of proteins from polyacrylamide gels to nitrocellulose sheets: procedure and some applications.. Proc Natl Acad Sci U S A.

[23] He LZ, Tribioli C, Rivi R, Peruzzi D, Pelicci PG (1997). Acute leukemia with promyelocytic features in PML/RARalpha transgenic mice.. Proc Natl Acad Sci U S A.

[24] Rego EM, Ruggero D, Tribioli C, Cattoretti G, Kogan S (2006). Leukemia with distinct phenotypes in transgenic mice expressing PML/RAR alpha, PLZF/RAR alpha or NPM/RAR alpha.. Oncogene.

[25] Gavish Z, Pinthus JH, Barak V, Ramon J, Nagler A (2002). Growth inhibition of prostate cancer xenografts by halofuginone.. Prostate.

[26] McGaha TL, Phelps RG, Spiera H, Bona C (2002). Halofuginone, an inhibitor of type-I collagen synthesis and skin sclerosis, blocks transforming-growth-factor-beta-mediated Smad3 activation in fibroblasts.. J Invest Dermatol.

[27] Nagler A, Ohana M, Shibolet O, Shapira MY, Alper R (2004). Suppression of hepatocellular carcinoma growth in mice by the alkaloid coccidiostat halofuginone.. Eur J Cancer.

[28] Sheffer Y, Leon O, Pinthus JH, Nagler A, Mor Y (2007). Inhibition of fibroblast to myofibroblast transition by halofuginone contributes to the chemotherapy-mediated antitumoral effect.. Mol Cancer Ther.

[29] van Dongen JJ, Macintyre EA, Gabert JA, Delabesse E, Rossi V (1999). Standardized RT-PCR analysis of fusion gene transcripts from chromosome aberrations in acute leukemia for detection of minimal residual disease. Report of the BIOMED-1 Concerted Action: investigation of minimal residual disease in acute leukemia.. Leukemia.

[30] Kogan SC, Ward JM, Anver MR, Berman JJ, Brayton C (2002). Bethesda proposals for classification of nonlymphoid hematopoietic neoplasms in mice.. Blood.

[31] Elkin M, Miao HQ, Nagler A, Aingorn E, Reich R (2000). Halofuginone: a potent inhibitor of critical steps in angiogenesis progression.. FASEB J.

[32] Leiba M, Cahalon L, Shimoni A, Lider O, Zanin-Zhorov A (2006). Halofuginone inhibits NF-kappaB and p38 MAPK in activated T cells.. J Leukoc Biol.

[33] Stecklair KP, Hamburger DR, Egorin MJ, Parise RA, Covey JM (2001). Pharmacokinetics and tissue distribution of halofuginone (NSC 713205) in CD2F1 mice and Fischer 344 rats.. Cancer Chemother Pharmacol.

[34] Kim SJ, Angel P, Lafyatis R, Hattori K, Kim KY (1990). Autoinduction of transforming growth factor beta 1 is mediated by the AP-1 complex.. Mol Cell Biol.

[35] Casini T, Pelicci PG (1999). A function of p21 during promyelocytic leukemia cell differentiation independent of CDK inhibition and cell cycle arrest.. Oncogene.

[36] Yagi K, Furuhashi M, Aoki H, Goto D, Kuwano H (2002). c-myc is a downstream target of the Smad pathway.. J Biol Chem.

[37] Derynck R, Akhurst RJ, Balmain A (2001). TGF-beta signaling in tumor suppression and cancer progression.. Nat Genet.

[38] Fortunel NO, Hatzfeld A, Hatzfeld JA (2000). Transforming growth factor-beta: pleiotropic role in the regulation of hematopoiesis.. Blood.

[39] Gnainsky Y, Kushnirsky Z, Bilu G, Hagai Y, Genina O (2007). Gene expression during chemically induced liver fibrosis: effect of halofuginone on TGF-beta signaling.. Cell Tissue Res.

[40] Zion O, Genin O, Kawada N, Yoshizato K, Roffe S (2009). Inhibition of transforming growth factor beta signaling by halofuginone as a modality for pancreas fibrosis prevention.. Pancreas.

[41] Sundrud MS, Koralov SB, Feuerer M, Calado DP, Kozhaya AE (2009). Halofuginone inhibits TH17 cell differentiation by activating the amino acid starvation response.. Science.

[42] Wu C, Wang S, Wang F, Chen Q, Peng S (2009). Increased frequencies of T helper type 17 cells in the peripheral blood of patients with acute myeloid leukaemia.. Clin Exp Immunol.

