# Supplementary figures and images for: Halofuginone Has Anti-Proliferative Effects in Acute Promyelocytic Leukemia by Modulating the Transforming Growth Factor Beta Signaling Pathway
Source: PLoS One. 2011 Oct 28;6(10):e26713. doi: 10.1371/journal.pone.0026713 (PMC3203897; doi:10.1371/journal.pone.0026713)

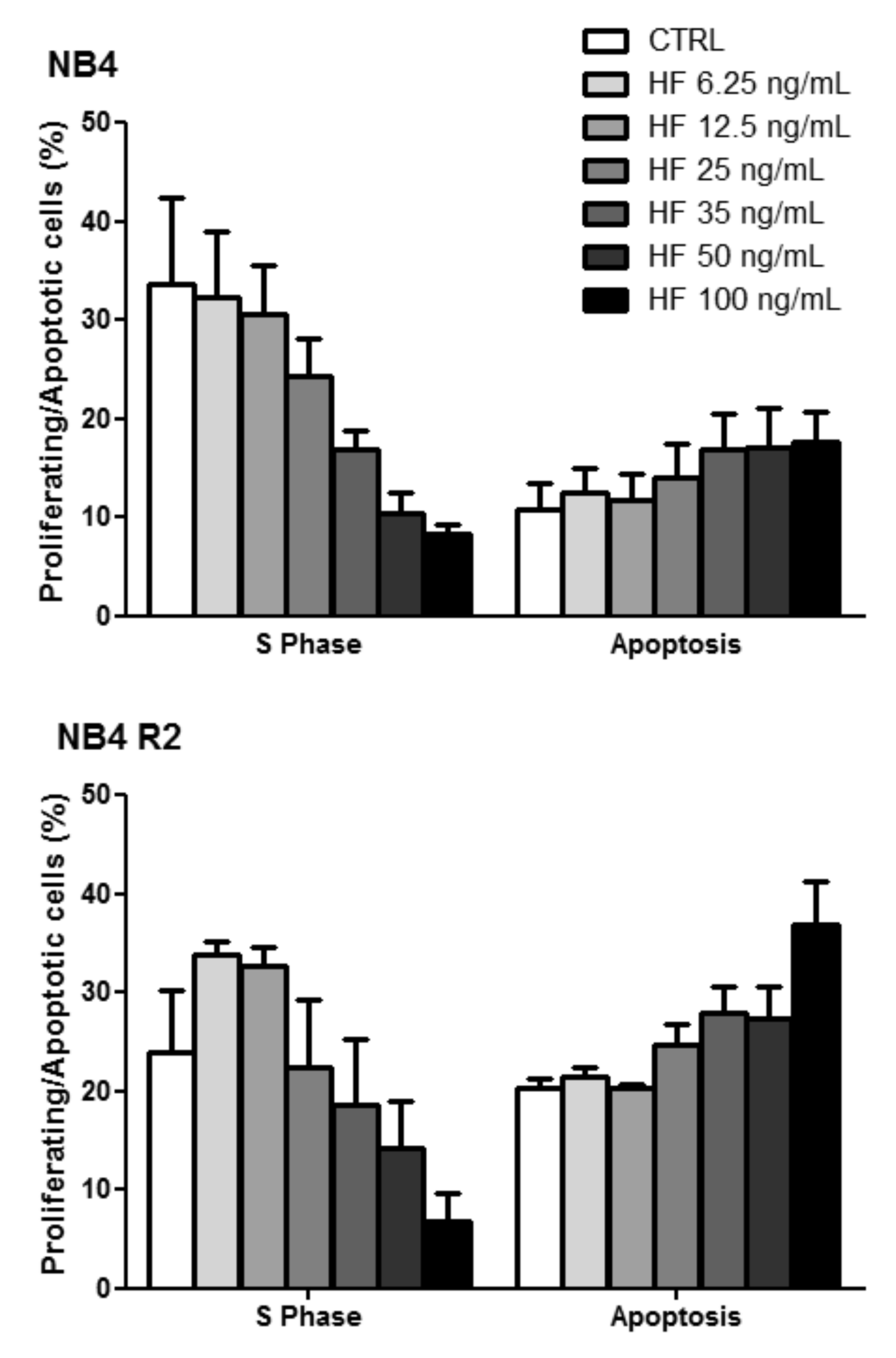

Supplement: Figure S1 — Cell cycle status of NB4 and NB4-R2 cells after treatment with increasing doses of halofuginone. Cell cycle status according to BrdU incorporation by NB4 (upper graphic) and NB4-R2 (lower graphic) cells after treatment with increasing doses of halofuginone. The multivariate analysis using the mixed linear model confirmed that, in both cell lines, the drug inhibited cell proliferation (P<0.001) and caused apoptosis (P = 0.002), although the pro-apoptotic effect in NB4 cells was visually less evident than the one observed in NB4-R2. (TIF) [file pone.0026713.s001.tif]
